# Feasibility and Safety of Laparoscopic Liver Resection for Hepatocellular Carcinoma with a Tumor Size of 5–10 cm

**DOI:** 10.1371/journal.pone.0072328

**Published:** 2013-08-21

**Authors:** Jun-hua Ai, Jian-wei Li, Jian Chen, Ping Bie, Shu-guang Wang, Shu-Guo Zheng

**Affiliations:** 1 Department of General Surgery, Chinese People’s Armed Police Force 8710 Hospital, Putian, People’s Republic of China; 2 The Institute of Hepatobiliary Surgery, Southwest Hospital, Third Military Medical University, Chongqing, People’s Republic of China; University Hospital of Essen, Germany

## Abstract

**Background:**

Although laparoscopic liver resection has developed rapidly and gained widespread acceptance for the treatment of benign liver diseases and hepatocellular carcinoma with a small tumor size, its usefulness for the treatment of large tumors is less clear, due to concerns about compromising oncological principles and patient safety. The purpose of this study was to explore the safety and feasibility of laparoscopic liver resection for the treatment of hepatocellular carcinoma with a tumor size of 5–10 cm.

**Methods:**

From March 2007 to December 2011, we performed liver resection in *275* patients with hepatocellular carcinoma with a tumor size of 5–10 cm. Laparoscopic liver resection was performed in 97 patients (Lap-Hx group) and open liver resection was performed in 178 patients (Open-Hx group). Operative time, estimated intraoperative blood loss, blood transfusion rate, and length of postoperative hospital stay were compared between the two groups. Early and intermediate-term postoperative outcomes were also compared.

**Results:**

Only one liver resection was performed for every patient with HCC in the present study.No operative deaths occurred in either group. Nine of the laparoscopic procedures were converted to open resection (conversion rate 9.28%). There were no significant differences in mean operative time (245±105 min vs 225±112 min; P = .469), mean estimated intraoperative blood loss (460±426 mL vs 454±365 mL; P = .913), or blood transfusion rate (4.6%, 4/88) vs (2.8%, 5/178)(P = .480) between the Lap-Hx and Open-Hx groups. However, postoperative hospital stay was shorter in the Lap-Hx group than the Open-Hx group (8.2±3.6 days vs 13.5±3.8 days; P = .028). There was a lower rate of postoperative complications in the Lap-Hx group than the Open-Hx group (9% vs 30%; P = .001), but there were no severe complications in either group. The median overall follow-up time was 21 months (range 2–50 months) and the median follow-up of time of survivors was 23 months. The median follow-up time was 25 months in the Lap-Hx group and 20 months in the Open-Hx group. The follow-up rate was 95% (84 patients) in the Lap-Hx group and 95% (169 patients) in the Open-Hx group, which was not a significant difference between the two groups (P = .20). Tumor recurrence occurred in 17 patients (20%) in the Lap-Hx group and 35 patients (21%) in the Open-Hx group, which was not a significant difference between the two groups (P = .876). A total of 33 patients (13%) died during the study period, including 12 patients (14%) in the Lap-Hx group and 21 patients (12%) in the Open-Hx group, which was not a significant difference between the two groups (P = .695). There were also no significant differences in the 1-year rates of overall survival (94% vs 95%; P = .942) or disease-free survival (93% vs 92%; P = .941), or the 3-year rates of overall survival (86% vs 88%; P = .879) or disease-free survival (66% vs 67%; P = .931), between the Lap-Hx and Open-Hx groups.

**Conclusions:**

Laparoscopic liver resection is safe and feasible in patients with hepatocellular carcinoma with a tumor size of 5–10 cm. Laparoscopic liver resection can avoid some of the disadvantages of open resection, and is beneficial in selected patients based on preoperative liver function, tumor size and location.

## Introduction

Laparoscopic liver resection (LLR) is an emerging technique in the field of hepatic surgery. Since LLR was first reported in 1991 [1], there has been an exponential increase in the number of reported LLR procedures. More than 3,000 LLR procedures have now been performed worldwide for the treatment of benign diseases and malignancy, and for living donor hepatectomy [2]. Although LLR was initially described for the treatment of small, peripheral, benign lesions, experienced teams are now safely performing more advanced LLRs including right hemihepatectomy, left hemihepatectomy, central hepatectomy, and extended right and left hepatectomy for the treatment of both benign and malignant lesions. Paralleling these advances in the technical feasibility of LLR, there is increasing acceptance of the minimally invasive approach for the treatment of larger tumors [3].

Numerous studies have clearly confirmed the safety and feasibility of LLR for the treatment of hepatocellular carcinoma (HCC) with a tumor size of ≤5 cm. The known benefits of laparoscopic surgery in general include: (1) smaller scars and reduced surgical trauma; (2) reduced need for narcotic pain relief, which facilitates early ambulation; (3) shorter hospital stay and earlier return to work; (4) reduced rate of complications such as ascites and liver failure, particularly in patients with liver cirrhosis and portal hypertension; (5) reduced physiological stress and effect on immunological function. The short-term outcomes of LLR are superior to those of open liver resection (OLR) for HCC with a tumor size of ≤5 cm, and intermediate-term and long-term outcomes are comparable between the two techniques [4], [5].

Kaneko et al [6] reported that LLR may be a suitable alternative to OLR for the treatment of HCC with a tumor size of ≤5 cm. However, LLR has been considered to be contraindicated in patients with HCC with a tumor size of ≥10 cm, because of concerns that the radical resection rate may be lower and the inherent limitations of the procedure. It is currently unclear whether LLR is suitable for the treatment of HCC with a tumor size of 5–10 cm, and few studies have compared LLR and OLR in these patients. It is therefore essential to further investigate the safety and feasibility of LLR for the treatment of HCC with a tumor size of 5–10 cm. In the present study, the clinical data of patients treated at our center of hepatobiliary surgery were retrospectively analyzed to confirm the feasibility of using LLR for the treatment of HCC with a tumor size of 5–10 cm, by evaluating parameters such as the rates of conversion to open surgery and complications. LLR and OLR were also compared to evaluate safety by analyzing survival and recurrence rates and the advantages of minimally invasive surgery.

## Materials and Methods

### Patients

After approval from our institutional review board, we retrospectively reviewed the medical records of 97 patients who underwent LLR (Lap-Hx group) and 178 patients who underwent OLR (Open-Hx group) from March 2007 to March 2011. The 178 patients in the Open-Hx group were considered potential candidates for LLR, but underwent OLR as a control group, with permission from the patients and their dependents. The preoperative HCC characteristics were similar between the Lap-Hx and Open-Hx groups. No other parameters except time period were matched between the two groups. Data were gathered from a retrospective review of medical charts with approval from the Institutional Review Board. This study was approved by the Southwestern Hospital ethics committees. The participants tested and their dependents have signed the written informed consent and provide their written informed consent to participate in this study. All clinical investigation have been conducted according to the principles expressed in the Declaration of Helsinki.

### Inclusion Criteria

All patients in this study had a preoperative diagnosis of HCC. Patients were selected for LLR based on the location of the tumor, the tumor’s proximity to major vascular structures, and the extent of resection required. Inclusion criteria for the Lap-Hx group were: (1) met the criteria for OLR; (2) tumor size of 5–10 cm; (3) no intrahepatic or distant metastasis; (4) no tumor thrombus in the portal vein, hepatic vein, vena cava, or bile duct; and no invasion of the diaphragm or surrounding tissues; (5) no rupture or bleeding of the tumor; (6) indocyanine green retention rate at 15 min of <15%, and a remnant liver volume/standard liver volume ratio of >50% in patients with liver cirrhosis and >35% in patients without liver cirrhosis; (7) Child-Pugh class A or B liver function; and (8) no previous upper abdominal surgery that absolutely contraindicated LLR. The contraindications for LLR were: (1) tumor size ≥10 cm, or tumor location that would interfere with intraoperative exposure and isolation of the hepatic hilum; (2) tumor encroaching on the hepatic hilum or the portal vein; (3) unable to tolerate a pneumoperitoneum; (4) severe upper abdominal adhesions; and (5) tumor adjacent to the major vascular structures.

Inclusion criteria for the Open-Hx group were: (1) met the criteria for LLR; and (2) patients and their dependents chose OLR after detailed explanation of the advantages and disadvantages of LLR and OLR by the surgeon. The 97 patients who underwent LLR were compared with the 178 patients who underwent OLR. All patients were treated according to the same standardized postoperative protocol aimed at early mobilization and feeding. This protocol starts oral intake on the first postoperative day and encourages early ambulation. Parameters for discharge include a full fluid diet, absence of ileus, ambulation, and adequate pain control with oral analgesia.

### Surgical Procedures

The surgical techniques used for LLR and OLR were similar, except for the method of abdominal access, and were based on previously described procedures [7], [8].

For LLR, patients were placed in the supine position under general anesthesia with endotracheal intubation. A CO_2_ pneumoperitoneum was established, with intra-abdominal pressure controlled at 12–14 mmHg (1 mmHg = 0.133 kPa). Five ports were usually used. The operating ports were placed in a fan-shape around the lesion. Based on individual characteristics, the operating table was tilted 15–45° to the right or left. The liver parenchymal transection plane was determined based on the findings of preoperative imaging, intraoperative exploration, intraoperative ultrasonography, and ischemic demarcation boundaries after hepatic vascular occlusion. The pattern of hepatic inflow occlusion was selected depending on the location of the lesion, the surgical approach, and the extent of liver cirrhosis. Selective hemihepatic inflow occlusion was used for anatomic hemihepatectomy. For lesions located in the left lateral lobe, right posterior lobe, or right anterior lobe, the intermittent Pringle maneuver was used if necessary, or the corresponding hepatic pedicle was dissected for selective regional occlusion. A harmonic scalpel was used to transect the liver parenchyma, in combination with other instruments such as the bipolar coagulator (Wolf Co., Inc., Germany), endoscopic rotation clip (Johnson and Johnson Co., Inc., USA), Hem-o-lok clip, and Endo-Linear Stapler (Johnson and Johnson Co., Inc., USA). Endoscopic ultrasonography (GE Co., Inc., USA) was performed to prevent deviation of the transection plane and ensure tumor-free margins. In patients with severe liver cirrhosis, irregular hepatectomy was performed with an incision margin of >1 cm whenever possible. The resected specimens were placed in a specimen bag and removed from the abdomen.

For OLR, briefly, all patients underwent epidural anesthesia followed by general anesthesia. Hemodynamic monitoring lines and vascular access lines were placed. Central venous pressure was maintained at approximately 5 cmH_2_O. A right subcostal incision was used with left subcostal extension if necessary. Following ultrasound examination, parenchymal transection was performed using a ring clamp. The procedures for vascular occlusion were similar to those for LLR, and the major vascular structures were ligated or sutured.

### Follow-Up

Patients received a follow-up telephone call from a clinical nurse to assess their postoperative condition. The follow-up regime after hepatic resection has been described elsewhere [9]. Briefly, patients underwent surveillance computed tomography every 3 months during the first year after resection, every 4 months during the second year, and every 6 months thereafter. If recurrence was suspected, patients also underwent ultrasonography and dynamic computed tomography, followed by angiography if recurrence was strongly suspected. This follow-up regime was strictly adhered to for the duration of the study period. Patients who tested positive for HBsAg were given postoperative anti-virus therapy according to our protocol.

### Statistical Analysis

Patient data including age, sex, presence or absence of chronic liver disease, tumor size, tumor location, type of resection, estimated intraoperative blood loss, operative time, length of postoperative hospital stay, morbidity, and mortality were compared between the two groups. Data are expressed as the mean ± standard deviation. Quantitative variables were compared using Fisher’s exact test and continuous variables were compared using the student’s t-test. A P value of <.05 was considered to be statistically significant. Statistical analyses were performed using the SPSS Statistical Software Package, version 18.0 (SPSS, Inc., Chicago, US).

## Results

All patients had a preoperative diagnosis of HCC based on imaging findings and pathological confirmation. There were no significant differences in age, gender, presence of cirrhosis, underlying pathology, presence of chronic liver disease, Child–Pugh score, or stage of the disease between the Lap-Hx and Open-Hx groups (Table 1).

**Table 1:** Characteristics of patients in the Lap-Hx and Open-Hx groups.

	Lap-Hx group (n = 97)	Open-Hx group (n = 178)	*P*
Age(years)			0.922
Mean	51.64	52.36	
Range	14–77	9–82	
Gender			0.177
Male	75	137	
Female	22	41	
Tumor diameter(cm)	7.85±2.15	7.64±2.36	0.829
Cirrhosis			0.211
Yes	78	143	
No	19	35	
Chronic liver disease			0.219
Type B hepatitis	75	136	
Alcoholic liver disease	18	37	
Child–Pugh			0.162
A	59	104	
B	38	74	
Histopathology			
Hepatocellular arcinoma	97	178	0.716
Stage(Okuda)			
I	63	116	0.274
II	34	62	

### Intraoperative Results

No operative deaths occurred in either group. There were no significant differences in mean operative time (245±105 min vs 226±112 min; P = .469), mean estimated intraoperative blood loss (460±426 mL vs 454±365 mL; P = .913), or blood transfusion rate (4.6% vs 2.8%; P = .480) between the Lap-Hx and Open-Hx groups. The mean resection margin was >1 cm in both groups, and there was no significant difference in the R0 resection rate between the two groups (P = .936) (Table II). However, the postoperative hospital stay was shorter in the Lap-Hx group than the Open-Hx group (8.2±3.6 days vs 13.5±3.8 days; P = .028) (Table 2). Nine of 97 LLRs were converted to OLR (conversion rate 9.3%). The reasons for conversion to OLR were shown in Table III. There was no evidence of intra- or postoperative gas embolism in any of the patients.

**Table 2:** Intraoperative parameters in the Lap-Hx and Open-Hx groups.

	Lap-Hx (n = 97)	Open-Hx group (n = 178)	*P*
Operative time(min)	245±105	225±112	0.469
Intraoperative blood loss(mL)	460±426	454±365	0.913
Rate of blood transfusion(%)	4.55%	2.81%	0.480
Resection margin size(cm)	1.53±0.59	1.36±0.62	0.818
R0 resection rate(%)	79.38	77.53	0.936
Postoperative hospital stay(d)	8.2±3.6	13.5±3.8	0.028

In the Lap-Hx group, 66 patients underwent regular hepatectomy and 34 patients underwent irregular hepatectomy. In the Open-Hx group, 129 patients underwent regular hepatectomy and 49 patients underwent irregular hepatectomy. There were no significant differences in the extent or type of resection between the two groups (Table 3). Details of the types of hepatectomy are shown in Table 4.

**Table 3:** Reasons for conversion of LLR to OLR (conversion rate 9.28%).

Reasons of conversion to OLR	Number of patients(n)
Uncontrollable bleeding	4
Transection parenchymal bleeding	1
Right hepatic vein bleeding	1
Right suprarenal vein bleeding	1
Tumor rupure bleeding	1
Positive incisional margin by laparoscopicviewing	2
Tumor encroaching on diaphragmatic muscle	3

**Table 4:** Types of resection performed in the Lap-Hx and Open-Hx groups.

	Lap-Hx group (n = 97)	Open-Hx group (n = 178)	*P*
Regular hepatectomy	64	129	0.462
Segment IV	1	12	*0.475*
Segment VI	17	16	*0.273*
Segment II + III	16	15	*0.451*
Segment V + VI	13	9	*0.808*
Segment VI + VII	2	18	*0.641*
Segment II + III + IV	8	15	*0.463*
Segment IV + V + VIII	2	31	*0.208*
Segment V + VI + VII + VIII	5	13	*0.645*
Irregular hepatectomy	24	49	*0.552*

### Early Postoperative Outcomes

There was a lower rate of postoperative complications in the Lap-Hx group than the Open-Hx group (11% vs 28%; P = .01). In the Lap-Hx group, complications with Clavien I, Clavien II accounted for 30% and 70%, respectively, and in Open-Hx group, complications with Clavien I, Clavien II and Clavien III accounted for 36%, 50% and 14%, respectively. Complications were less severe in the Lap-Hx group than the Open-Hx group. Complications in the Lap-Hx group included pulmonary infection (n = 3), local pulmonary atelectasis (n = 1), collections (n = 2), pleural effusion (n = 1), infection of the incisional wound (n = 1), intra-abdominal infection and abscess (n = 1), and renal dysfunction (n = 1). Complications in the Open-Hx group included pulmonary infection (n = 9), local pulmonary atelectasis (n = 2), collections (n = 7), pleural effusion (n = 8), ascites (n = 4), infection of the incisional wound (n = 5), intra-abdominal infection and abscess (n = 4), urinary tract infection (n = 3), renal dysfunction (n = 1), incisional hernia (n = 2), and bile leakage (n = 5). Details of the postoperative complications are shown in Table 5.

**Table 5:** Postoperative complications in the Lap-Hx and Open-Hx groups according to the Clavien system.

Complication	Lap-Hx group (n = 97)	Open-Hx group (n = 178)	P
**Clavien I**	3	18	0.000
Renal dysfunction	1	1	0.985
Ascites		4	0.003
Infection of incisional wound	1	5	0.010
Pleural fluid	1	8	0.000
**Clavien II**	7	25	0.003
Urinary tract infection		3	0.010
Pulmonary infection	3	9	0.004
Local pulmonary atelectasis	1	2	0.326
Intra-abdominal infection and abscess	1	4	0.032
Collections	2	7	0.007
**Clavien III**		7	0.002
Bile leakage		5	0.001
Incisional hernias		2	0.036

### Late Postoperative Outcomes

**Figure 1:**
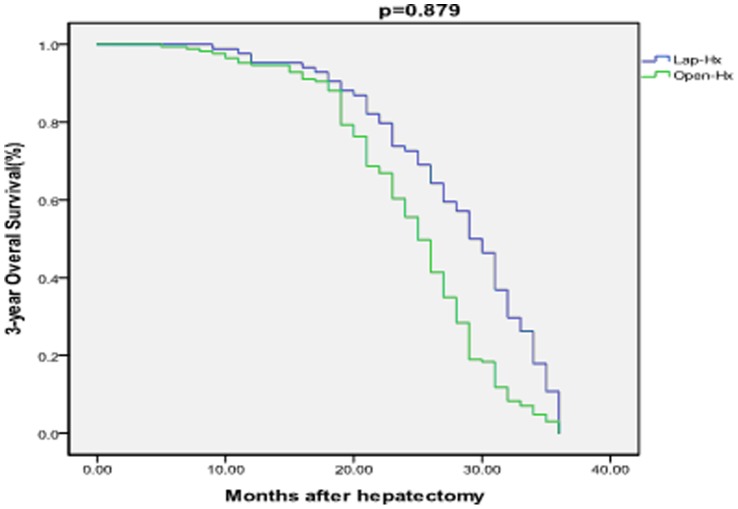
Comparison of 3-year overall survival rates between the Lap-Hx and Open-Hx groups. There was no significant difference in the 3-year overall survival rate between the two groups. There was no significant difference in the 3-year overall survival rate between the two groups.

**Figure 2:**
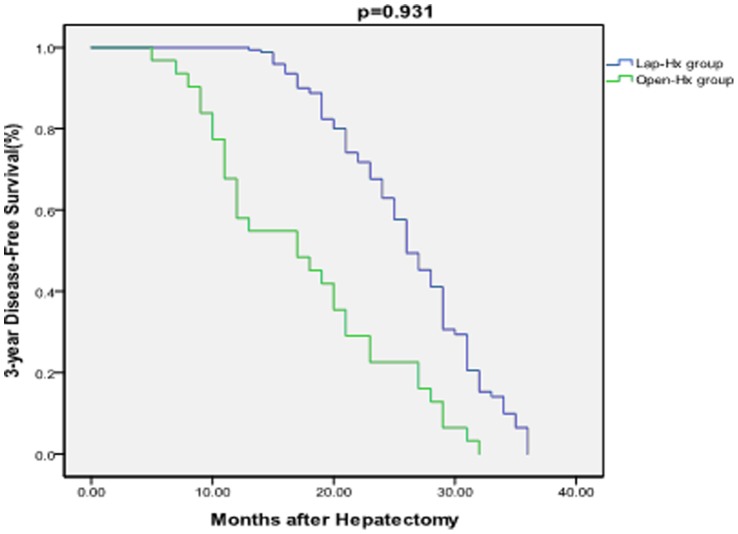
Comparison of 3-year disease-free survival rates between the Lap-Hx and Open-Hx groups. There was no significant difference in the 3-year disease-free survival rate between the two groups. There was no significant difference in the 3-year disease-free survival rate between the two groups.

The median overall follow-up time was 21 months (range 2–50 months), and the median follow-up time of survivors was 23 months. The median follow-up time was 27 months in the Lap-Hx group and 25 months in the Open-Hx group. The follow-up rate was 96% (84 patients) in the Lap-Hx group and 95% (169 patients) in the Open-Hx group, which was not a significant difference between the two groups (P = .20). Recurrence of HCC occurred in 17 patients (20%) in the Lap-Hx group and 35 patients (21%) in the Open-Hx group, which was not a significant difference between the two groups (P = .876). A total of 33 patients (13%) died during the study period, comprising 12 patients (14%) in the Lap-Hx group and 21 patients (12%) in the Open-Hx group, which was not a significant difference between the two groups (P = .695). The causes of death included recurrence of tumor, liver failure, renal failure, gastrointestinal bleeding, severe pulmonary infection and pulmonary failure. There were no significant differences between the Lap-Hx and Open-Hx groups in the 1-year rates of overall survival (94% vs 95%; P = .942) or disease-free survival (93% vs 92%; P = .941), or in the 3-year rates of overall survival (86% vs 88%; P = .879) or disease-free survival (66% vs 67%; P = .931) (Figure 1 and 2). In the Open-Hx group, there were no cases of local recurrence in the hepatic stump. No metastases were detected in the abdominal cavity or at the laparoscopy port sites in any of the patients. In patients with recurrence of HCC, transhepatic arterial chemoembolization and/or radiofrequency ablation was performed.

## Discussion

LLR has been widely and rapidly accepted in the field of hepatobiliary surgery because laparoscopic surgery is associated with a reduced need for narcotic pain relief, shorter hospital stay, earlier return to work, and less physiological stress than the open abdominal surgery [5]. The appeal of LLR is easy to appreciate, but it is a technically challenging procedure as expertise in both laparoscopy and open liver surgery are essential. Liver parenchymal transection carries the risk of massive hemorrhage and bile leakage, both of which can be challenging to manage laparoscopically. In addition, patients requiring liver resection often have underlying liver cirrhosis, further complicating parenchymal transection. The current reports of LLR mainly describe the treatment of benign tumors and small HCCs, and there is ongoing debate regarding the use of LLR for the treatment of malignant tumors and large HCCs [10].

HCC with a tumor size of >10 cm has been considered to be a contraindication for LLR because of concerns that the radical resection rate may be lower, and the inherent limitations of the operative procedure. Takagi et al [11]suggested that it was preferable to use LLR only for tumors measuring <6 cm in diameter. As few studies have compared LLR and OLR for the treatment of HCC with a tumor size of 5–10 cm, there is still uncertainty about the safety and feasibility of using LLR in these patients. Technical advances have allowed LLR to be performed safely, but equivalent recurrence and survival rates must also be verified before LLR can be considered a suitable alternative to OLR.

Most HCC patients in the present study had concurrent liver cirrhosis. It is more difficult to perform hepatectomy for HCC in patients with liver cirrhosis than without. In patients with liver cirrhosis, uncontrolled bleeding may occur during liver dissection or parenchymal transection. However, LLR for HCC has successfully been performed in patients with liver cirrhosis. The results of the present study show no differences in operative time, mean estimated intraoperative blood loss, or blood transfusion rate between the Lap-Hx and Open-Hx groups, indicating that intraoperative blood loss can be controlled effectively during LLR even in patients with liver cirrhosis. LLR for HCC is therefore safe and feasible in selected patients. Our results are consistent with those of previous studies [12].

We found that the operative time tended to be longer in the Lap-Hx group than the Open-Hx group. This finding may be related to the learning curve for LLR for the resection of larger tumors. In the present study, the extent and type of resection were similar between the two groups. Only 9 of 97 LLR procedures were converted to OLR. The rate of conversion to OLR was higher in our center than other institutes because the tumor size was larger and the rate of patients combined with liver cirrhosis was higher in the present study.

It is of interest that the postoperative hospital stay was significantly shorter in the Lap-Hx group than the Open-Hx group. Compared with OLR, LLR can also reduce the need for narcotic pain relief, reduce postoperative physiological stress, and contribute to earlier return to work in patients with HCC. There was a significantly lower rate of postoperative complications in the Lap-Hx group than the Open-Hx group, with the increased complication rate in the Open-Hx group primarily due to pulmonary infection, atelectasis, reactive pleural effusion, ascites, intra-abdominal infection and ascites, urinary tract infection, incisional hernia, and wound infections. Complications in the Lap-Hx group were primarily due to pulmonary infection and collections in the hepatic stump. Pulmonary infection and atelectasis, reactive pleural effusion and ascites, and wound infections contributed to the development of hypoproteinemia in patients with liver cirrhosis in the Open-Hx group, which prolonged recovery time and hospitalization. According to the Clavien classification [13],the rate of postoperative complications and the severity of complications were lower in the Lap-Hx group than the Open-Hx group. This reasons for this may include the more extensive manipulation of intra-abdominal organs, longer incision, more severe pain, delayed ambulation, and decreased postoperative cough and expectoration in the Open-Hx group. In the present study, the percentage of bile leakage after Open-Hx was very low and there was no bile leakage after laparoscopic approach, which was concerned with some measures that we took to reduce the percentage of bile leakage during operating.For example, after transecting the liver parenchyma, to identify whether it existes bile leakage or not, we use the white absorbent gauzes to compress the liver cross-section and observe them for several minutes. If the white absorbent gauzes changed into the flavous, we will suture and ligate the liver cross-section till the white absorbent gauzes keep white. Moreover, there are many superiority of LLR for treating HCC, including magnifying visual field, clear operative field, showing tiny structure of bile duct and blood vessel clearly, and carrying out corresponding treatment, consequently in abstracto, the incidence rate of bile leakage is lower in the LLR groups than the OLR groups.

There is concern that the lack of tactile sensation and distance perception, and the limited visual field during transection of the liver parenchyma, may affect the resection margin and the rate of radical resection in LLR for HCC. However, there were no significant differences in resection margin status, or 1- or 3-year overall or disease-free survival rates, between the Lap-Hx and Open-Hx groups in the present study, indicating that these concerns did not affect outcomes. The possible reasons for this include our strict patient selection criteria, careful preoperative and intraoperative evaluation, and use of intraoperative ultrasonography. There were no recurrences near the hepatic stump after LLR, and no cases of port-site recurrence or tumor seeding were detected.

The first hepatectomies for the large HCC were reported by Hüscher et al [14]. Widespread use of laparoscopic hepatectomy for the large HCC has been hindered by fears of major hemorrhage and the technical challenges of portal, caval, and hepatic vein dissection. Recently, Dagher et al [15] reported a prospective study of 210 laparoscopic major liver resections (136 right and 74 left hepatectomies) at five medical centers (two in Europe, two in the United States, and one in Australia) from 1997 to 2008. Their results showed that a purely laparoscopic approach was used in 43.3% of cases, whereas a hand-assisted approach was used in 56.7% of cases. Conversion to laparotomy was required in 12.4% of cases. The mortality rate was 1% and the specific morbidity rate (hemorrhage, ascites, or biloma) was 8.1%. In patients with malignant disease, negative margins (R0 resections) were achieved in 97.4%. Comparison of the early experience (n = 590) with the late experience (n = 5120) found that operative time, blood loss, portal clamping time, conversion rate, and length of hospital stay all improved over time. The investigators concluded that laparoscopic major hepatectomy was feasible in selected patients, if performed by surgeons who were already well experienced in laparoscopic minor hepatectomy [16], [17]. In other reported studies, Belli et al [18] found that the laparoscopic resection group had a significantly wider resection margin than the open resection group, whereas Kevin et al [4] found no difference in the margin-free resection rate between laparoscopic and open liver resection.

In conclusion, LLR for HCC with a tumor size of 5–10 cm is safe and feasible, even in appropriately selected patients with liver cirrhosis. With continued development and use of LLR worldwide, the superiority of LLR over OLR will become increasingly apparent. LLR is a superior technique because it is minimally invasive, and it has been shown to have favorable outcomes such as a shorter postoperative hospital stay, reduction of postoperative pain and ileus, and less effect on immunological function than OLR [19]. However, there is insufficient evidence to determine if LLR is more suitable than OLR for the treatment of HCC. Although our study enables us to draw some preliminary conclusions, the reliability of these conclusions is limited because of the relatively small sample size and the limitations of the study design. A well-designed, randomized controlled trial with large sample size, such as a multi-center trial, with standardized liver resection techniques and measurements, would achieve more definitive conclusions.
